# Anthelmintic activity on *Haemonchus contortus* and toxicity of benzoyl-carvacrol: a study *in vitro*, *in silico* and *in vivo*

**DOI:** 10.1590/S1984-29612025009

**Published:** 2025-03-14

**Authors:** Andreza Pereira Braga, José Vilemar de Araújo, Matheus Luiggi Freitas Barbosa, Raphael Ferreira Oliveira, Daniela Ribeiro Alves, Wildson Max Barbosa da Silva, Matheus Nunes da Rocha, Emmanuel Silva Marinho, Márcia Machado Marinho, Wesley Lyeverton Correia Ribeiro, Selene Maia de Morais, Claudia Maria Leal Bevilaqua, Lorena Mayana Beserra de Oliveira

**Affiliations:** 1 Programa de Pós-graduação em Ciências Veterinárias, Faculdade de Veterinária, Universidade Estadual do Ceará – UECE, Fortaleza, CE, Brasil; 2 Programa de Pós-graduação Ciências Naturais, Centro de Ciências e Tecnologia, Universidade Estadual do Ceará – UECE, Fortaleza, CE, Brasil; 3 Centro de Ciências Exatas e Tecnologia, Universidade Estadual do Vale do Acaraú – UVA, Sobral, CE, Brasil; 4 Departamento de Fisiologia e Farmacologia, Faculdade de Medicina, Universidade Federal do Ceará – UFC, Fortaleza, CE, Brasil

**Keywords:** Carvacrol, benzoylation, β-tubulin, toxicological safety, gastrointestinal nematodes, Carvacrol, benzoilação, β-tubulina, segurança toxicológica, nematoides gastrintestinais

## Abstract

Carvacrol is isolated from essential oils and possesses activity against gastrointestinal nematodes of small ruminants. Benzoylation has been proposed to improve its pharmacological and pharmacokinetic properties. The objectives of this study were to evaluate the ovicidal activity of benzoyl-carvacrol (BC) against *Haemonchus contortus*, the *in silico* interaction of BC with the β-tubulin protein and the toxicity of this compound. Carvacrol was subjected to benzoylation and analyzed by gas chromatography coupled to mass spectrometry (GC/MS). The activity of BC and carvacrol was evaluated against *H. contortus* in the egg hatching test. The *in silico* study was based on molecular docking with the β-tubulin and thiabendazole used as control. The acute toxicity test was performed with BC and carvacrol by up-and-down procedure (limit test: 2,000 mg/kg) in Wistar rats. GC/MS confirmed the benzoylation. BC and carvacrol inhibited egg hatching by 99.70 and 98.89% at concentrations of 3.16 and 1 mg/mL, respectively, and interacted with β-tubulin. No mortality was caused by compounds, but rats treated with carvacrol demonstrated intoxication signs. These findings indicated that BC showed effect on *H. contortus* and can potentially interact with β-tubulin of nematodes in addition to presenting toxicological safety in laboratory animals.

## Resumo

O carvacrol é isolado de óleos essenciais e possui atividade contra nematoides gastrintestinais de pequenos ruminantes. A benzoilação tem sido proposta para melhorar suas propriedades farmacológicas e farmacocinéticas. Os objetivos deste estudo foram avaliar a atividade ovicida do benzoil-carvacrol (BC) contra *Haemonchus contortus*, a interação *in silico* do BC com a proteína β-tubulina e a toxicidade desse metabólito. O carvacrol foi submetido à benzoilação e analisado por cromatografia gasosa, acoplada à espectrometria de massas (CG/MS). A atividade anti-helmíntica do BC e carvacrol foi avaliada no teste de eclosão de ovos, usando *H. contortus*. O estudo *in silico* foi baseado no *docking* molecular com β-tubulina, e o tiabendazol foi o controle. O teste de toxicidade aguda do BC e carvacrol foi realizado pelo procedimento *up-and-down* (limite testado: 2.000 mg/kg) em ratos Wistar. CG/MS confirmou a benzoilação. BC e carvacrol inibiram a eclosão dos ovos em 99,70 e 98,89% nas concentrações de 3,16 e 1 mg/mL, respectivamente, e interagiram com a β-tubulina. Os compostos não causaram mortalidade, entretanto ratos tratados com carvacrol demonstraram sinais de intoxicação. Esses achados indicaram que BC apresentou efeito sobre *H. contortus* e pode, potencialmente, interagir com a β-tubulina, além de apresentar segurança toxicológica em animais de laboratório.

## Introduction

The gastrointestinal nematodes (GIN) reduce animal productivity and reproductive performance and leads to losses in sheep and goat farming (Chagas et al., 2022). In tropical and subtropical countries, *Haemonchus contortus* is known as the most pathogenic GIN of small ruminants. This parasite feeds on blood in the abomasum and causes anemia, lack of appetite, lethargy, weight loss, dehydration and edema, leading to death in severely affected animals (Basripuzi et al., 2020).

The control of GIN is based on the administration of synthetic anthelmintics (AH). However, the frequent and inappropriate use of these drugs has led to failures in their effectiveness, culminating in a worldwide problem of anthelmintic resistance (Salgado & Santos, 2016). The essential oils (EO) extracted from plants and their bioactive compounds can offer an alternative strategy to reduce dependence on chemoprophylaxis in sheep and goats (Sebai et al., 2021).

Carvacrol (5-isopropyl-2-methylphenol) is a phenolic monoterpene present in several EO from aromatic plants of the family Lamiaceae, including the genus *Origanum* and *Thymus* (Baser, 2008). This secondary metabolite has several pharmacological actions. Considering that carvacrol is a phenolic compound, it is more toxic than their esters, as carvacryl acetate (CA), which was synthesized by our research group to obtain a semisynthetic derivative with an improved pharmacological profile and low toxicity. Acetylation is the process of replacing the hydrogen atom of the hydroxyl radical with the acetyl group. It was demonstrated that CA showed *in vitro* and *in vivo* anthelmintic activity and was less toxic than carvacrol (André et al., 2016).

The benzoylation is another strategy that has been applied to enhance pharmacological activity and reduce the toxicity of compounds. This structural modification is a chemical reaction that is carried out by replacing the hydrogen of the hydroxyl radical with the benzoyl group (Morais et al., 2014). However, the benzoyl-carvacrol (BC) evaluation on *H. contortus* and its toxicity has not yet been demonstrated. Furthermore, few studies have provided evidence about the probable mechanisms of action of compounds against *H. contortus*. In this sense, *in silico* strategies are very useful complementary tools that help in the understanding of interaction of bioactive compounds with the biological target (Borges et al., 2023), including the β-tubulin protein of GIN (Rahal et al., 2022).

Thus, the aims of this preliminary study were to investigate the anthelmintic activity of BC against *H. contortus* eggs, evaluate the interaction of this carvacrol derivative against β-tubulin protein by molecular docking and determine its toxicological safety in rats.

## Materials and Methods

### Benzoylation reaction of carvacrol

**Figure 1:**
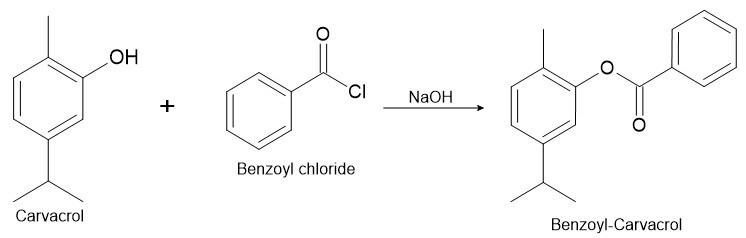
Carvacrol benzoylation process.

BC was obtained through benzoylation of carvacrol (Sigma-Aldrich^®^, USA) (Figure 1). For this synthesis, carvacrol (7.5 g) was dissolved in 40 mL of a cold solution of 5% sodium hydroxide (NaOH), with subsequent addition of benzoyl chloride (7 g). This solution was homogenized for 20 minutes by stirring until consumption of all benzoyl chloride. After this period the pH of the solution was adjusted to be neutral and extracted with chloroform (3 x 15 mL). The chloroform phases were joined, washed with water then dried with sodium sulfate, and evaporated to dryness for obtaining a semisolid product (Morais et al., 2014). The formation of benzoylated product was accompanied by thin layer chromatography (TLC).

### Analysis of benzoyl-carvacrol

The chemical structure of the BC was confirmed by gas chromatography coupled to mass spectrometry (GC/MS) using a GCMS model QP2010S (Shimadzu^®^, Japan). The following experimental conditions were employed: Rtx-5MS capillary column (5% diphenyl/95% dimethylpolysiloxane) with dimensions of 30 m × 0.25 mm × 0.25 µm df; helium carrier gas (24.2 mL/min, in constant linear speed mode); an initial temperature of 250 °C, in split mode (1:100) and detector temperature of 250 °C. The programmed column temperature was from 35 to 180 °C, at 4 °C/min, from 180 to 280 °C, at 17 °C/min, and then maintained at 280 °C for 10 min. Mass spectra were obtained at 70 eV of electron impact. The sample was injected in a volume of 1 µL. The compound was identified by relative retention time to known compounds and comparing it to compounds in the National Institute of Standards and Technology database.

### Anthelmintic test

A lamb infected monoespecifically with *H. contortus* was used as an egg donor of this nematode to perform the egg hatching test (EHT). The *H. contortus* Kokstad isolate used is resistant to benzimidazoles, levamisole and macrocyclic lactones (Fauvin et al., 2010). The EHT was performed according to the technique described by Coles et al. (1992). Suspensions (250 μL) containing approximately 100 fresh eggs were incubated with the same volume of solutions from the following treatments: G1 – 0.59 to 3.16 mg/mL BC; G2 – 0.06 to 1 mg/mL carvacrol; G3 – 1.5% Tween 80 as negative control and G4 – 0.2 mg/mL thiabendazole (TBZ) as positive control. Carvacrol and TBZ were purchased from Sigma-Aldrich^®^. The eggs were incubated for 48 h to 25 °C, and drops of 5% iodine solution were added to stop the egg hatching. Then eggs and L1 were counted in a light microscope. For each treatment and controls, three repetitions with five replicates were performed.

### Computational methods

Molecular docking studies were carried out with BC against β-tubulin protein (Rahal et al., 2022). The phylogenetic position of *H. contortus* concerning the free-living nematode *Caenorhabditis elegans* was considered to select the structure of β-tubulin from the Protein Data Bank (PDB ID: 6E88). In the target preparation step, residues were removed, polar hydrogens were added, and Gasteiger charges were calculated using the Autodocktools™ code.

The generated grid parameters were centralized to involve the entire structure of the β-tubulin using the axes (239.398 x, 88.188 y, 205.431 z), size (24.655 x, 18.084 y, 24.261z) and exhaustiveness 64. The simulations were performed with the control TBZ – (PubChem CID5430) to obtain comparative data. The TBZ is the main standard synthetic AH representative of the benzimidazoles class. Fifty simulations of molecular docking were performed. Each simulation generated 20 poses, which were later selected based on the Root Mean Square Deviation (RMSD) statistical parameter with values up to 2.0 Å (Yusuf et al., 2008) and affinity energy less than -6.0 kcal/mol (Shityakov & Foerster, 2014) for selecting the best pose.

### Toxicity test

The acute toxicity test in rats was performed to define the toxicological safety profile for BC and carvacrol. Twelve female Wistar rats with an average weight of 120 ± 2.5 g were allowed to acclimatize to the experimental conditions (luminosity: 12 h/12 h, light/dark; temperature: 22 ± 2 °C; relative humidity: 60%) for seven days in polypropylene boxes. Commercial feed for rodents (Nuvilab^®^, Brazil) and filtered water were provided *ad libitum*.

The acute toxicity was performed according to Organisation for Economic Co-operation and Development (OECD, 2022) – Test Guideline No. 425 (Acute Oral Toxicity: Up-and-Down Procedure). The BC and carvacrol were administered orally in a single dose progression (175; 440; 1,100 and 2,000 mg/kg). Each animal was carefully evaluated for up to 48 h before the decision of the dose to be administered to the next animal and further observed until day 14 post administration. Signs of toxicity as changes in skin and fur, eyes and mucous membranes, and also respiratory, circulatory, autonomic and central nervous systems, and somatomotor activity and behavior pattern were observed. Dosing was discontinued when five reversals occurred in six consecutive animals treated with BC or carvacrol. All decisions on controlled doses and estimated lethal dose for 50% (LD_50_) of rats were determined by OECD software AOT425StatPgm using a limit dose of 2,000 mg/kg and a sigma value of 0.4.

### Statistical analysis

In EHT, the percentage of effectiveness of BC and carvacrol on egg hatching was determined according to the following Formula 1:


$$\left(number\;of\;hatched\;larvae/number\;of\;hatched\;larvae\;+\;number\;of\;eggs\right)\;x\;100$$ (1)

The inhibition effects at each concentration of treatments were analyzed using analysis of variance (ANOVA) and compared by Tukey's test (P<0.05). All analyzes were performed using GraphPad Prism^®^ 8.0.1 software. The effective concentration of BC and carvacrol to inhibit 50% (EC_50_) of egg hatching was determined by probit linear regression using SPSS^®^ 22.0 for Windows.

## Results

The GC/MS analysis confirmed the benzoylation reaction and the BC was the major component. The effect of BC and carvacrol on the hatching of *H. contortus* eggs is shown in Table 1. BC and carvacrol showed ovicidal activity. The highest two concentrations inhibited more than 96% of egg hatching. These results did not differ statistically from the positive control (TBZ). Both compounds had a dose-dependent effect. The EC_50_ values for BC and carvacrol were 1.11 and 0.16 mg/mL, respectively.

**Table 1:** Mean efficacy (± standard deviation) of benzoyl-carvacrol (BC) and carvacrol on *Haemonchus contortus* egg hatching.

Concentration (mg/mL)	Benzoyl-carvacrol (BC)	Concentration (mg/mL)	Carvacrol
3.16	99.70 ± 0.30^Aa^	1	98.89 ± 0.55^Aa^
2.37	96.77 ± 1.28^Aa^	0.5	98.01 ± 1.01^Aa^
1.58	89.45 ± 5.96^Ba^	0.25	91.97 ± 2.85^Ba^
0.79	24.68 ± 4.97^Ca^	0.12	36.66 ± 5.11^Cb^
0.59	15.30 ± 2.97^Da^	0.06	10.71 ± 1.89^Db^
Tween 80	2.93 ± 0.60^Ea^	Tween 80	2.35 ± 0.48^Ea^
Thiabendazole	100 ± 0.00^Aa^	Thiabendazole	100 ± 0.00^Aa^

The capital letters indicate comparisons of the means in the columns, and the lowercase letters denote comparisons of the means in the rows. Different letters indicate significantly different values (P < 0.05).

**Figure 2:**
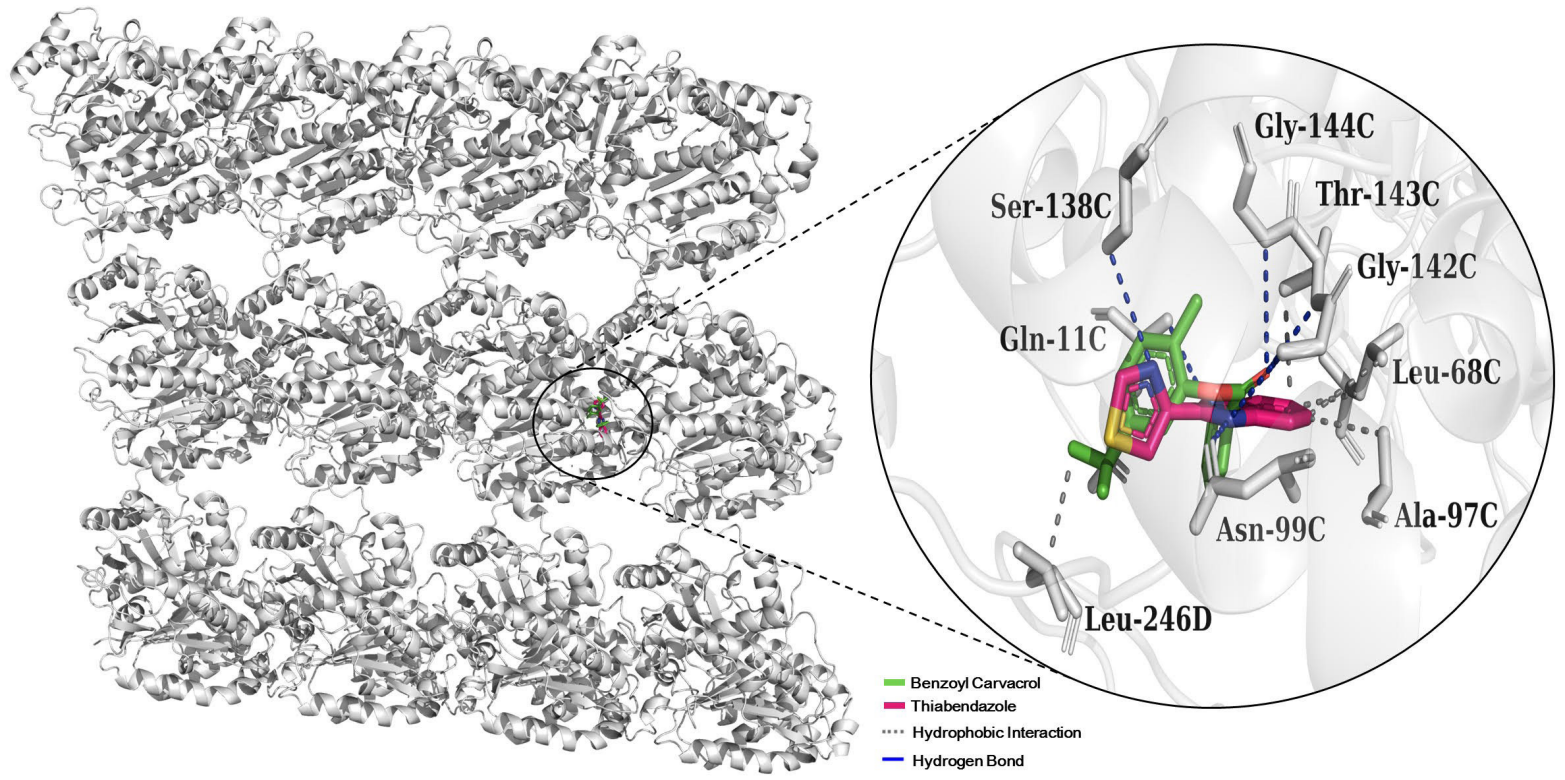
Interaction complex between the ligands benzoyl-carvacrol (BC) and thiabendazole (TBZ) with β-tubulin.

The molecular docking simulations generated RMSD values of 1.993 Å for BC and 1.243 Å for TBZ. The affinity energy values for BC and TBZ were -5.9 and -6.4 kcal/mol, respectively. Figure 2 demonstrates the interaction standards of β-tubulin and ligands (BC and TBZ). The β-tubulin/BC complex is predominantly formed by hydrophobic interactions involving residues Gln 11C (3.48 Å), Gln 11C (3.54 Å) and Leu 246D (3.60 Å) and three strong hydrogen bonds with Gly 142C (2.59 Å), Thr 143C (2.00 Å) and Gly 144C (3.07 Å). The TBZ/β-tubulin complex is formed by four hydrophobic interactions with Leu 68C (3.61 Å), Leu 68C (3.74 Å), Ala 97C (3.69 Å) and Thr 143C (3.56 Å) and four strong hydrogen bonds with residues Gln 11C (3.02 Å), Asn 99C (2.08), Ser 138C (3.02 Å) and Gly 142C (2.61 Å). The analysis of interactions with amino acid residues of β-tubulin showed that BC binds to close but a different site than the control (TBZ).

In the acute toxicity test, no mortality or signs of intoxication or behavioral change were observed in the rats treated with BC. No mortality also was observed during the test period in the carvacrol-treated group. However, rats that received a dose of 2,000 mg/kg carvacrol showed behavioral changes, such as agitation and self-cleaning with their paws in the mouth area immediately after administration. Furthermore, weight loss was observed for 3 days in animals that received doses of 1,100 and 2,000 mg/kg of carvacrol, but after this period the animals began to gain weight. Therefore, the estimated LD_50_ of BC and carvacrol was greater than 2,000 mg/kg.

## Discussion

The anthelmintic effect of different monoterpenes was evaluated on eggs of a multidrug-resistant *H. contortus* isolate. Carvacrol (EC_50_ = 0.11 mg/mL) was more effective than thymol (EC_50_ = 0.13 mg/mL), linalool (EC_50_ = 0.29 mg/mL), eugenol (EC_50_ = 0.57 mg/mL), vanillin (EC_50_ = 0.57 mg/mL), cineole (EC_50_ = 4.74 mg/mL) and limonene (EC_50_ = 207.56 mg/mL) (Katiki et al., 2017). Despite these promising results, carvacrol was structurally modified through acetylation to enhance its activity and reduce its toxicity (André et al., 2016). However, another type of structural modification on carvacrol, as benzoylation, had not yet been evaluated for its efficacy against *H. contortus* and toxicity.

CA showed an ovicidal effect (EC_50_ = 1.70 mg/mL) lower than its original monoterpene, carvacrol (EC_50_ = 0.17 mg/mL). Furthermore, the acetylated derivative of the monoterpene thymol (EC_50_ = 1.90 mg/mL) showed lower ovicidal activity than the thymol (EC_50_ = 0.08 mg/mL). These data indicate that acetylation did not potentiate the ovicidal activity of these monoterpenes (André et al., 2016, 2017). In the present study, the EC_50_ of BC was 1.11 mg/mL and EC_50_ of carvacrol was 0.16 mg/mL. These data demonstrated that benzoylation was more effective than acetylation.

The carvacrol ovicidal effect could be attributed to the phenolic group in its chemical structure. This radical possibly inhibits enzymes (proteases, lipases, chitinases, betaglucosidase and leucine aminopeptidase) that are responsible for inhibiting hatching of eggs (Molan & Faraj, 2010). This mechanism is similar to the polyphenols or tannins that have the hydroxyl radical in their chemical structure (Vargas-Magaña et al., 2014).

The esterification of hydroxyl group found in carvacrol may provide different pharmacological and pharmacokinetic properties and, in this work, the benzoylation caused decrease in ovicidal activity of carvacrol against *H. contortus*. Previous studies of carvacrol and its ester derivative (CA) have shown better results when evaluated in larval development test and adult worm motility test using this nematode (André et al., 2016). Then, other studies can be formed to evaluate if the BC can show better efficacy than carvacrol when tested with other parasite stages.

Recently, computational approaches have attracted considerable interest due to their potential to accelerate drug discovery in terms of time, staffing and costs and to find action mechanism of compounds (Shaker et al., 2021). Molecular docking is an *in silico* technique that allows studying the interaction model between the target (protein) and the ligand (compound) through calculations. Based on the distance and binding energy between the ligand and the binding sites, interaction models and affinities can be calculated to simulate optimal interactions (Bielska et al., 2011).

In molecular docking, the target selected was β-tubulin. The β-tubulin is only known target of AH available in protein repositories, as Protein Data Bank. This protein is the target of benzimidazoles. The action mechanism of benzimidazoles as TBZ is well elucidated and related to the inhibition of egg hatching in gastrointestinal nematodes. According to Köhler (2001), these compounds inhibit the polymerization of the parasite microtubules by binding to β-tubulin. The benzimidazoles inhibit the embryonation and eggs hatching of nematode by interfering with the formation of microtubules (Coles et al., 1992). Consequently, the eggs of the parasites do not develop correctly and become non-viable, reducing the parasitic load and preventing new reinfections. Therefore, β-tubulin is a potential target for new anthelminthic compounds. The correct choice of target to be used in *in silico* studies is the step to ensure that the target-ligand interaction represents the action of the compound that leads to the biological effect observed during experimental assays (Borges et al., 2023). It was observed that BC had affinity energy against β-tubulin similar to TBZ with the Gibbs free energy interval (ΔG) < -6.0 kcal/mol (Shityakov & Foerster, 2014).

The OECD oral toxicity method (Guideline 425) allows the estimation of an LD_50_, and the results enable a substance to be classified according to the Globally Harmonized System (GHS) for the classification of chemicals that cause acute toxicity (OECD, 2022). In the present study, BC and carvacrol LD_50_ was greater than 2,000 mg/kg. According to GHS (United Nations, 2011), substances with an LD_50_ of 2,000 to 5,000 mg/kg are classified in category 5, the category of substances that present a relatively low risk of acute toxicity. However, the carvacrol caused behavior changed in rats indicating toxicity. These results confirm that the benzoylation process reduces the toxicity of its parent monoterpene (carvacrol). As the toxicological safety of BC is not known, the acute oral toxicity test in laboratory animal model was performed to define its safe dose for further administration in sheep. BC was more efficient on eggs of *H. contortus* and less toxic than CA (André et al., 2016). Thus, there will be no toxicity problem if there is a need to increase the BC dose in tests with sheep. As for the residues of this compound in meat and milk, they should be clarified in future studies.

To our knowledge, this preliminary study is the first that synthetized the BC and assessed the anthelmintic activity and toxicity of this derivative of carvacrol. The results indicate that BC may affect the egg hatching of *H. contortus.* Molecular docking studies indicate that the BC may interact with important amino acid residues of β-tubulin, likely serving as its probable mechanism of action. Furthermore, the BC demonstrated toxicological safety in rats and tests using larvae and adults of *H. contortus* should also be carried out to evaluate the effect on these life stages for further evaluation in lambs and goats.
